# Study on the Regulatory Effect of Water Extract of *Artemisia annua* L. on Antioxidant Function of Mutton Sheep via the Keap1/Nrf2 Signaling Pathway

**DOI:** 10.3390/antiox14070885

**Published:** 2025-07-18

**Authors:** Gen Gang, Ruiheng Gao, Ruizhen Li, Xiao Jin, Yuanyuan Xing, Sumei Yan, Yuanqing Xu, Binlin Shi

**Affiliations:** College of Animal Science, Inner Mongolia Agricultural University, Hohhot 010018, China; ganggen@emails.imau.edu.cn (G.G.); gaoruiheng@emails.imau.edu.cn (R.G.); 13633328345@emails.imau.edu.cn (R.L.); yaojinxiao@imau.edu.cn (X.J.); xingyuanyuan2014@163.com (Y.X.); yansmimau@imau.edu.cn (S.Y.)

**Keywords:** water extracts of *Artemisia annua* L., mutton sheep, antioxidative index, Keap1/Nrf2 pathway, peripheral blood lymphocytes

## Abstract

This study was conducted through in vivo and in vitro experiments and aimed to reveal the regulatory effect of water extract of *Artemisia annua* L. (WEAA) on the antioxidant function of mutton sheep and the underlying mechanism. In the in vivo experiment, 32 Dorper × Han female sheep (3 months old; avg. body weight: 24 ± 0.09 kg) were allocated to four groups (eight lambs/group) and fed a diet containing 0, 500, 1000, and 1500 mg/kg WEAA, respectively. In the in vitro experiments, peripheral blood lymphocytes (PBLs) were cultured with different doses of WEAA (0, 25, 50, 100, 200, 400 µg/mL) to determine the optimal concentration, followed by a 2 × 2 factorial experiment with four treatment groups (six replicates per treatment group): the ML385(−)/WEAA(−) group, the ML385(−)/WEAA(+) group, the ML385(+)/WEAA(−) group, and the ML385(+)/WEAA(+) group. The results showed that WEAA supplementation dose-dependently increased serum, liver and spleen tissue total antioxidant capacity, glutathione peroxidase (GSH-Px), and catalase (CAT) activity while reducing malondialdehyde level (*p* < 0.05). Moreover, WEAA supplementation significantly upregulated the liver and spleen expression of *nuclear factor erythroid 2-related factor 2*, *superoxide dismutase 2*, *GSH-Px*, *CAT* and *NAD(P)H quinone dehydrogenase 1* (*p* < 0.05) while significantly downregulating the *kelch-like ECH associated protein 1* expression in a dose-dependent manner (*p* < 0.05), thereby activating the Keap1/Nrf2 pathway with the peak effect observed in the 1000 mg/kg WEAA group. Additionally, supplementation with 100 µg/mL of WEAA had significant antioxidation activity in the culture medium of PBLs. Its action mechanism involved the Keap1/Nrf2 pathway; specifically, WEAA exerted its antioxidant effect by upregulating the gene expression related to the Keap1/Nrf2 pathway. In conclusion, WEAA enhances sheep’s antioxidant capacity by up-regulating Keap1/Nrf2 pathway genes and boosting antioxidant enzyme activity. The results provided experimental support for the potential application of WEAA in intensive mutton sheep farming.

## 1. Introduction

With the rapid advancement of the intensive breeding process in China, mutton sheep breeding is confronted with challenges from a series of stressors [1]. These stressors, including the high-density rearing environments, environmental pollution, imbalanced feed nutrition, and frequent diseases, are closely related to the oxidation imbalance and immune dysregulation in the sheep organism [2,3]. These processes have adverse effects on livestock health and performance [4]. A growing body of evidence indicates that plant-derived feed supplements can effectively regulate the cellular antioxidant activity of domestic animals [5,6]. Plants and their extracts can effectively regulate reactive oxygen species (ROS) levels by modulating the processes of ROS production and clearance, as well as enhancing redox homeostasis capacity, activating relevant signaling pathways such as Keap1/Nrf2, PI3K/AKT, MAPK and TLR4/NF-κB, promoting the growth and functional specialization of immune cells, and ensuring the normal oxidative balance of the organism [7,8,9]. Plant-derived bioactive constituents, including flavonoids and polysaccharides, exhibit both relative non-toxicity and minimal side effects, making them promising candidates for natural antioxidant development [10]. They can activate the signal transduction pathways of immune cells and effectively promote crucial antioxidant enzymes activity, such as superoxide dismutase (SOD), glutathione peroxidase (GSH-Px), catalase (CAT), etc., playing a crucial promoting role in the processes of antioxidation and anti-inflammation [11], which provides an effective strategy to address the problems faced with sheep breeding.

**Figure 1:**
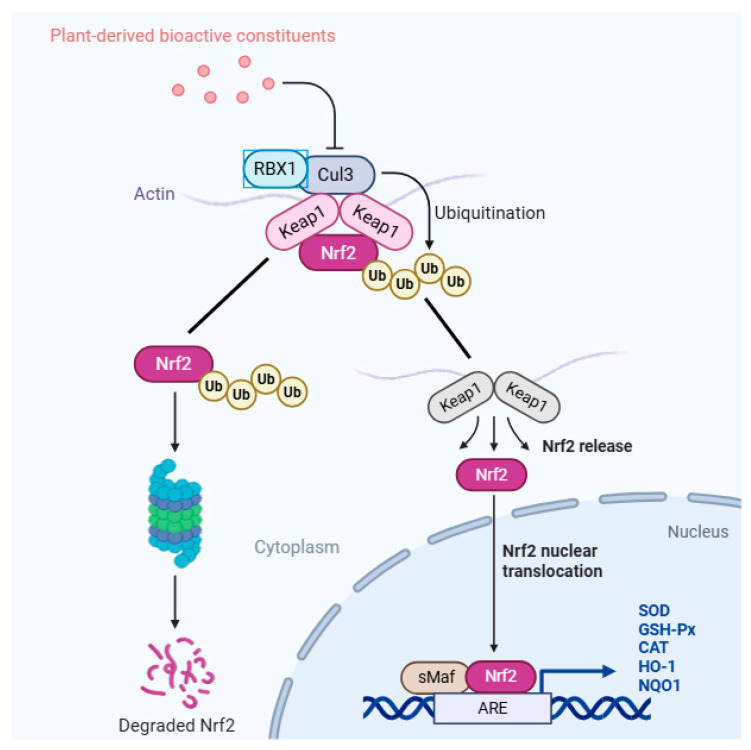
The schematic of the Keap1/Nrf2 signaling pathway modulated by plant-derived bioactive constituents. This pathway is hypothesized to underlie the antioxidant effects of WEAA in sheep, as explored in this study.

*Artemisia annua* L. (*A. annua*, Sweet Annie) is traditionally renowned for its antimalarial properties; however, modern research has discovered multiple pharmacological effects, including antibacterial, antiviral, anticancer, anti-inflammatory, and antioxidant effects [12]. Notably, *A. annua* and its extracts have been shown to eliminate free radicals and enhance antioxidant enzyme activity in both in vitro and in vivo studies, thereby improving oxidative balance and enhancing resistance to foreign pathogens [13]. Animal studies have yielded encouraging evidence of the beneficial impacts of *A. annua* extracts on oxidative stress parameters. For instance, dietary addition with *A. annua* extract was shown to enhance the liver antioxidant capacity and physiological parameters in *Micropterus salmonids* [14]. In rat models, the *A. annua* leaf extract further protected against oxidative damage by up-regulating antioxidant enzymes and reducing biomarkers of lipid peroxidation [15]. Moreover, recent findings suggested that the protective actions of *A. annua* involved the modulation of signaling pathways associated with cell survival and antioxidant responses, such as the PI3K/Akt/Nrf2 axis [16]. Nuclear factor erythroid 2-related factor 2 (Nrf2) is a crucial regulatory factor for intracellular antioxidation and anti-inflammatory damage [17]. After dissociating from Kelch like ECH associated protein 1 (Keap1), Nrf2 localizes to nuclei and complexes with the antioxidant response element (ARE), promoting the transcription of cell-protective factors and subsequently upregulating the expression of related antioxidant genes. This process leads to a marked enhancement within the intracellular environment of antioxidant enzymes, thereby scavenging free radicals and ROS and resisting oxidative damage [18]. Moreover, the activation of the Nrf2/ARE signaling pathway not only has an antioxidant stress effect but also enhances the immune response of the organism [19]. Therefore, the Keap1/Nrf2 signaling pathway, as a key protective pathway, has significant biological significance for the health of the organism (Figure 1).

Although the research on *A. annua* and its extracts in terms of antioxidant effects is relatively widespread, systematic investigations into the effects of the water extract of *A. annua* (WEAA) on the antioxidant function in sheep remain conspicuously absent, with a notable paucity of targeted inquiries. Considering the wide distribution of *A. annua* in Inner Mongolia, it is extremely necessary to conduct its rational development and utilization. Therefore, this study was conducted to first to assess firstly the antioxidant activity of WEAA in the serum, liver and spleen of sheep and then to further systematically verify its mechanism of action by incorporating in vitro Nrf2 pathway blockade experiments on lymphocytes. This approach will provide an innovative theoretical framework facilitating the scientific utilization of *A. annua* in the field of sheep breeding.

## 2. Materials and Methods

All animal procedures were approved by the Institutional Animal Care and Use Committee (Approval No. NND2021097, approved on 5 November 2021) and conducted in accordance with the National Standard Operating Procedures (GB/T 43562-2023, *Code of Practice for Livestock and Poultry Slaughtering Operation*—*Sheep*, China) [20].

### 2.1. Preparation of Water Extract of Artemisia annua *L.* (WEAA)

WEAA was prepared from *A. annua* aerial parts collected from Hohhot, Inner Mongolia, China, in August 2021. The production procedure involved drying the plant material in the shade; cutting it into short segments and extracting with water (1:25 *w*/*v*) at 80 °C for 7 h; filtering, collecting the filtrate, concentrating, and freeze-drying; and preparing the powder. All samples were maintained at 4 °C to ensure stability. The chemical ingredients of WEAA are listed in Supplementary Table S1.

### 2.2. Animals and Grouping

All animals enrolled in this study (purchased from Livestock Farm, Ordos City, Inner Mongolia Autonomous Region, China) were determined to have a normal health status.

Prior to the commencement of the experiment, 32 female Dorper × Han sheep, each around 3 months old, with a body weight of 24 ± 0.09 kg, were randomly allocated into four experimental groups, with eight animals per group. The control group was fed a standard basal diet only. The remaining groups received the basal diet amended with WEAA at doses of 500, 1000, and 1500 mg/kg, respectively. The basal diet was formulated in alignment with the Nutrient Requirements of Meat-type Sheep (NY/T 816-2021) [21], ensuring it met the animals’ dietary needs. Detailed information on the diet’s ingredients and nutrient composition is provided in Table 1.

### 2.3. Raising and Management

The feeding experiment lasted for 75 days (with a 15-day adaptation and a 60-day formal trial period). During the adaptation phase, sheep underwent deworming and stomach-strengthening procedures while being fed a basal diet. They received two feedings daily at 8:00 and 16:00 h, and it was ensured that they had more than 5% of feed left each day. During the experimental period, sheep were individually housed in pens (pen size: 2.4 m × 3.6 m) within a naturally ventilated barn with windows. Equipped with individual feeders, the sheep had ad libitum access to both feed and water, and all were managed under consistent conditions. Daily health monitoring was conducted for every animal to promptly detect any abnormalities, ensuring the integrity of experimental data and the well-being of the sheep throughout the study.

### 2.4. Collection of Peripheral Blood and Preparation of Lymphocyte Suspension

The collection and suspension preparation of peripheral blood lymphocytes (PBLs) were performed as described previously by Xing et al. (2024) [8]. In this experiment, blood samples were obtained from the external jugular vein of 3 healthy Dorper × Small Tail Han sheep (female, 3 months old, body weight 26 ± 3.5 kg) using standard venipuncture techniques (sterile heparin sodium anticoagulant blood collection tube) at 08:00 a.m., following a 10 h fasting period, and ad libitum access to water and a balanced pelleted diet for sheep. Subsequently, 5 mL of the blood samples was transferred to 10 mL sterile tubes, and an equal volume of PBS diluent (Tianjin Haoyang Biological Products Technology Co., Ltd., TBD, Tianjin, China) was added and thoroughly mixed. Next, 5 mL of sheep lymphocyte separation medium (TBD, China) was aliquoted into 15 mL centrifuge tubes, and an equal volume of the diluted blood sample was slowly overlaid along the tube wall to form a clear interface above the separation medium, which was centrifuged at 375× *g* for 20 min using a density gradient centrifugation device. Then, the milky white lymphocyte layer in the middle was carefully aspirated into a new centrifuge tube and washed twice with PBS washing solution (TBD, China). After washing, all PBLs were suspended in RPMI-1640 medium (10% FBS and 1% Pen-Strep). Finally, the Cell Drop Fluorescence/Brightfield Fully Automated Cell Counter (DeNovix Inc., Wilmington, DE, USA) was used to count the PBLs, and the cell concentration of the PBLs suspension was adjusted to 1 × 10^6^ cells/mL for the subsequent in vitro lymphocyte culture experiment.

### 2.5. Cell Culture and WEAA Treatment

The in vitro experiments included two trials to explore the antioxidant effect of WEAA on PBLs and its underlying mechanism.

Trial 1: Optimization of WEAA concentration focused on examining the antioxidant capacity of WEAA on PBLs to determine the optimal concentration. This investigation made use of a single-factor randomized block design to ensure the validity of the results. The cultivated PBLs were randomly split into 6 treatment groups. In the treated PBLs, WEAA was added at concentrations of 0, 25, 50, 100, 200, and 400 μg/mL, with six replicates for each treatment. The PBLs suspension with an adjusted concentration was inoculated into a 24-well sterile culture plate (1.8 mL PBLs suspension per well), and the working solutions of different concentrations were added, respectively. The final volume of the culture system in each well was 2 mL. The culture was incubated in a 37 °C, 5% CO_2_ incubator for 24 h. Then, the culture plates were taken out and operated aseptically on a super clean bench. From each well, 100 μL of the PBL suspension was extracted and placed on a 96-well sterile culture plate. Subsequently, 10 μL of CCK-8 assay reagent (Mechem Express Inc., MCE, Monmouth Junction, NJ, USA) was added to each culture well, and the culture was continued for 3 h to determine the cell viability. The remaining PBL suspension was collected in a centrifuge tube and centrifuged at 210× *g* for 10 min at 4 °C. Then, the supernatant fluids were gathered and frozen at −20 °C for the determination of antioxidant-related indicators in the PBL culture medium. At the same time, the cells were harvested and frozen at −80 °C to extract the total RNA in the cells to quantify the expression levels of genes implicated in antioxidant functions in the PBLs.

Trial 2: Exploration of the regulatory mechanism via the Keap1/Nrf2 signaling pathway aimed to investigate whether WEAA regulated the antioxidant capacity of PBLs through the Keap1/Nrf2 signaling pathway. A 2 × 2 factorial experiment design was adopted to conduct a cross-group design of two factors. The two independent variables were ML385 (MCE Inc., USA) (two levels: added and not added) and WEAA (two WEAA addition levels: added and not added). Four treatment groups were set up, with 6 replicates in each treatment: the ML385(−)/WEAA(−) group (control group, cultured for 36 h, no special treatment), theML385(−)/WEAA(+) group (normally cultured for 12 h, then added to 100 µg/mL WEAA and cultured for 24 h), the ML385(+)/WEAA(−) group (added to 10 µmol/mL ML385, incubated for 12 h, then continued to culture for 24 h), and the ML385(+)/WEAA(+) group (added to 10 µmol/mL ML385, incubated for 12 h, then added to 100 µg/mL WEAA and cultured for 24 h).

### 2.6. Collection of Sheep Serum, Liver, and Spleen Samples

During the experimental feeding period, blood was collected from the jugular vein of sheep before morning feeding on days 0, 30, and 60 of the experiment. The samples were centrifuged at 1200× *g* for 15 min at 4 °C. The serum was obtained and stored at −20 °C for future use.

On day 60 of the experiment, the experimental sheep fasted for 12 h and were slaughtered at a commercial slaughterhouse in compliance with the National Standard Operating Procedures, and the abdominal cavity was opened. The liver and spleen tissues were immediately collected and stored at −80 °C for future analysis. An appropriate amount of each tissue sample was taken, and the thawed tissue samples were homogenized with precooled 0.9% physiological saline (centrifuged at 1500× *g* for 15 min at 4 °C). The supernatant was obtained and placed at −80 °C for subsequent analysis.

### 2.7. Cell Viability

The cell viability of PBLs was assessed using the CCK-8 cell proliferation-toxicity assay. PBLs were treated with WEAA at doses of 0, 25, 50, 100, 200, and 400 μg/mL (6 replicates per treatment group) in 96-well plates. After 3 h of incubation, the absorbance at 450 nm was determined by means of a Bio-Rad (Hercules, CA, USA) microplate reader, and cell viability was subsequently calculated based on the optical density (OD) values.

### 2.8. Antioxidant Indices in Serum, Liver, and Spleen

The levels of T-AOC (ABTS method, Kit A015-2-1, 734 nm), T-SOD (WST-1 method, Kit A001-3-2, 450 nm), GSH-Px (DTNB colorimetric method, Kit A005-1-2, 412 nm), CAT (molybdenum acid method, Kit A007-1-1, 405 nm), and malondialdehyde (MDA) (TBA method, TBARS assay, Kit A003-1-2, 532 nm) were measured spectrophotometrically using a V-1000 UV-Vis spectrophotometer (Shanghai MUPUDA Instruments Co., Ltd., Shanghai, China) with commercial assay kits following the manufacturer’s instructions (Nanjing Jiancheng Bioengineering Institute, Nanjing, China).

### 2.9. RNA Extraction and Real-Time Quantitative PCR (qRT-PCR) Analysis

After extracting total RNA from spleen tissue, the total RNA was reverse-transcribed to cDNA using the Sens quest Lab Cycler Gradient PCR Instrument in combination with the cDNA Kit (Yeasen Bio-technology Co., Ltd., Shanghai, China) in accordance with the instructions. The specific primer sequences targeting the genes of interest (GOIs) and reference genes (*β-actin* and *GAPDH*) are detailed in Table 2. The mRNA expression levels were quantified using qRT-PCR according to the 2^−ΔΔCt^ method.

### 2.10. Statistical Analysis

Initial data processing was performed using Microsoft Excel 2021, followed by statistical analysis to assess linear and quadratic effects on various response indices as a function of increasing WEAA levels using the regression analysis procedure of SAS Version 9.4. A variance analysis of the 2 × 2 factorial design data was conducted, in which the two independent variables were ML385 (two levels: added and not added) and WEAA (two WEAA addition levels: added and not added), and the individual effects, main effects of the two treatment factors, and the interaction effects between the factors were statistically analyzed, respectively. Subsequently, one-way ANOVA with Duncan’s multiple range test was applied to compare specific differences among WEAA treatment groups. Results are presented as means with the corresponding SEM. The threshold for statistical significance was set at *p* ≤ 0.05, while probability values falling within the range of 0.05 ≤ *p* < 0.10 were interpreted as suggesting a trend.

## 3. Results

### 3.1. Effects of WEAA on Antioxidant Indexes of Sheep

As shown in Table 3, the antioxidant indexes in the serum exhibited no significant changes among treatments on day 0 (*p* > 0.10).

On day 30, with the increase in dietary WEAA supplementation, the serum CAT activity showed a significant linear or quadratic rise (*p* < 0.01); the serum GSH-Px activity tended to increase linearly or quadratically (*p* = 0.060; *p* = 0.068); the MDA contents tended to decrease linearly or quadratically in the serum (*p* = 0.076; *p* = 0.084). Compared with the control group, dietary 500, 1000, and 1500 mg/kg of WEAA increased the serum CAT activity (*p* < 0.05).

On day 60, with increased WEAA supplementation, the serum T-AOC demonstrated a linear or quadratic rise (*p* < 0.05); GSH-Px activity exhibited a linear or quadratic rise (*p* < 0.05), and MDA content decreased quadratically (*p* < 0.05); CAT activity significantly increased linearly or quadratically (*p* < 0.01). Compared with the control group, dietary 1000 and 1500 mg/kg of WEAA increased serum T-AOC (*p* < 0.05); a diet with 1000 mg/kg of WEAA increased GSH-Px activity (*p* < 0.05) and decreased MDA contents (*p* < 0.05); and a diet containing 500, 1000 and 1500 mg/kg of WEAA increased CAT activity (*p* < 0.01).

Table 4 shows the liver and spleen antioxidant indexes. With the increase in dietary WEAA supplementation, the liver CAT activity showed a quadratic increase (*p* < 0.05), while the MDA levels in the liver decreased quadratically (*p* < 0.05). In addition, compared with the control group, dietary 50 and 1000 mg/kg of WEAA increased CAT activity (*p* < 0.05); a diet with 500 mg/kg of WEAA decreased liver MDA levels (*p* < 0.05).

The results of spleen antioxidant indexes showed that as dietary WEAA supplementation increased, the spleen T-AOC significantly increased quadratically (*p* < 0.01); the T-SOD activity demonstrated a linear or quadratic increasing trend (*p* = 0.092; *p* = 0.056) and the activity of GSH-Px demonstrated a significant quadratic rise (*p* < 0.05); the spleen CAT activity significantly increased quadratically (*p* < 0.01); and the MDA levels in the spleen significantly decreased quadratically (*p* < 0.01). Compared with control group, a diet containing 500 and 1000 mg/kg of WEAA increased T-AOC levels (*p* < 0.05), and decreased MDA content (*p* < 0.05) in the spleen; a diet with 1000 mg/kg of WEAA increase activity of GSH-Px (*p* < 0.05) in the spleen; and a diet containing 500, 1000 and 1500 mg/kg of WEAA increased spleen CAT activity (*p* < 0.05).

### 3.2. Effects of WEAA on the Expression of Antioxidant-Related Genes in the Liver and Spleen of Sheep

**Figure 2:**
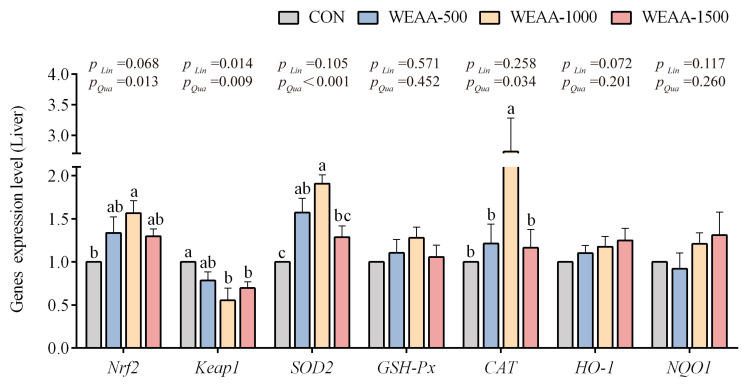
Effect of dietary WEAA levels on the expression of genes related to antioxidation in the liver of sheep. Note: WEAA: Water extracts of *Artemisia annua* L. CON: Control; WEAA-500: 500 mg/kg WEAA; WEAA-1000: 1000 mg/kg WEAA; WEAA-1500: 1500 mg/kg WEAA; values are expressed as the means of 8 sheep in each group. Dose-dependent effects of WEAA (Lin: Linear; Qua: Quadratic); ^a–c^ Different letters indicate significant differences between mean values for a given indicator (*p* < 0.05), whereas the probability value of 0.05 ≤ *p* < 0.10 was considered as a tendency (±standard error).

Figure 2 shows that with the increase in dietary WEAA supplementation, the liver gene expression of *Nrf2* showed a linear increasing trend (*p* = 0.068) and a significant quadratic increase (*p* < 0.05); the expression of *Keap1* decreased in a linear or quadratic manner (*p* < 0.05; *p* < 0.01); the expression of *SOD2* and *CAT* showed a quadratic increase (*p* < 0.01; *p* < 0.05). Compared with control group, dietary supplementation with 1000 mg/kg of WEAA levels increased the liver gene expression of *Nrf2* and *CAT* (*p* < 0.05); a diet with 500 and 1000 mg/kg of WEAA increased the expression of *SOD2* (*p* < 0.05); and a diet with 1000 and 1500 mg/kg of WEAA decreased expression of *Keap1* in the liver (*p* < 0.05).

**Figure 3:**
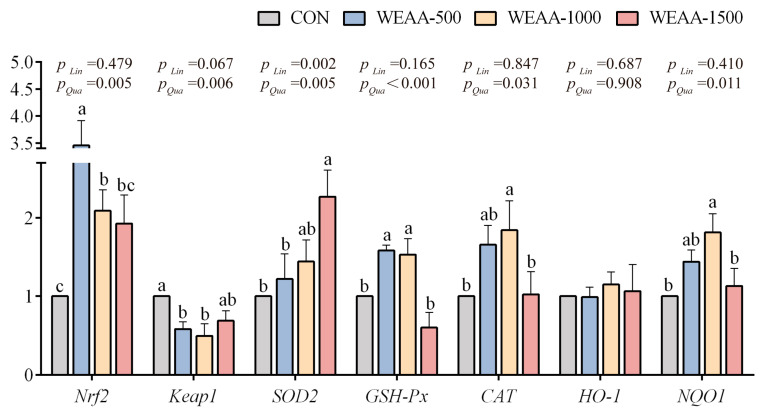
Effect of dietary WEAA levels on the expression of genes related to antioxidation in the spleen of sheep. Note: WEAA: Water extracts of *Artemisia annua* L. CON: control; WEAA-500: 500 mg/kg WEAA; WEAA-1000: 1000 mg/kg WEAA; WEAA-1500: 1500 mg/kg WEAA; values are expressed as the means of 8 sheep in each group. Dose-dependent effects of WEAA (Lin: Linear; Qua: Quadratic); ^a–c^ Different letters indicate significant differences between mean values for a given indicator (*p* < 0.05), whereas the probability value of 0.05 ≤ *p* < 0.10 was considered as a tendency (±standard error).

Similar to the trends in the liver, Figure 3 shows that with the increase in dietary WEAA supplementation, the spleen gene expression of *Nrf2*, *GSH-Px*, *CAT* and *NQO1* exhibited a quadratic rise (*p* < 0.05); the gene expression of *SOD2* demonstrated a significant linear or quadratic rise (*p* < 0.01); the expression of *Keap1* showed a linear decreasing trend (*p* = 0.067) and a significant quadratic decline (*p* < 0.01). Compared with the control group, a diet containing 500 and 1000 mg/kg of WEAA significantly elevated the *Nrf2* and *GSH-Px* gene expression levels (*p* < 0.05) and decreased the gene expression of *Keap1* (*p* < 0.05) in the spleen; a diet with 1500 mg/kg of WEAA increased the expression of *SOD2* (*p* < 0.05); and a diet with 1000 mg/kg of WEAA significantly elevated the gene expression of *CAT* and *NQO1* in the spleen (*p* < 0.05).

### 3.3. Effects of WEAA on the Cell Viability of PBLs

**Figure 4:**
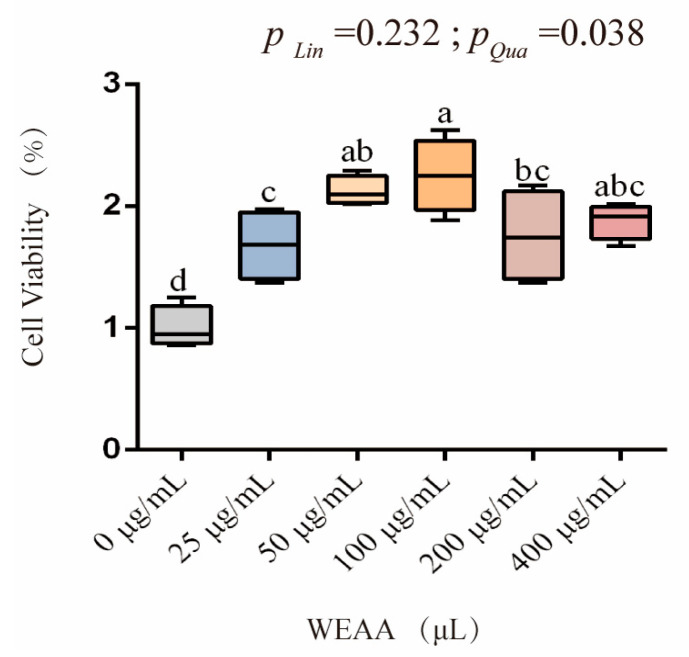
Effects of WEAA on cell viability of PBLs. Note: WEAA: Water extracts of *Artemisia annua* L. Different concentrations of WEAA (0, 25, 50, 100, 200, and 400 μg/mL); values are expressed as the means of 6 replicates in each group. Dose-dependent effects of WEAA (Lin: Linear; Qua: Quadratic); ^a–d^ Different letters indicate significant differences between mean values for a given indicator (*p* < 0.05), whereas the probability value of 0.05 ≤ *p* < 0.10 was considered as a tendency (±standard error).

The effect of WEAA on the viability of PBLs is shown in Figure 4. As can be seen from the figure, with the increase in the addition level of WEAA, the viability of PBLs exhibited a significant quadratic increasing effect (*p* < 0.05).

### 3.4. Effects of WEAA on Antioxidant Indexes in Culture Medium of PBLs

Table 5 illustrates the effects of WEAA on antioxidant indexes in the culture medium of PBLs. With the increase in the addition level of WEAA, the PBLs’ T-AOC and CAT activity in culture medium of PBLs showed a linear increasing trend (*p* = 0.085; *p* = 0.078) and a significant quadratic rise (*p* < 0.01; *p* < 0.05); the T-SOD and GSH-Px activity exhibited a significant quadratic increase (*p* < 0.01; *p* < 0.01).

Compared with the control group, the groups supplemented with 100 and 200 µg/mL of WEAA showed an increase in the T-AOC and T-SOD activity (*p* < 0.05) in the medium of PBLs; supplementation with 50–200 µg/mL of WEAA increased the activity of GSH-Px (*p* < 0.05) in the medium of PBLs; supplementation with 50 and 100 µg/mL of WEAA increased PBLs CAT activity in the medium of PBLs (*p* < 0.05).

### 3.5. Effects of WEAA on the Expression of Antioxidant-Related Genes of PBLs

**Figure 5:**
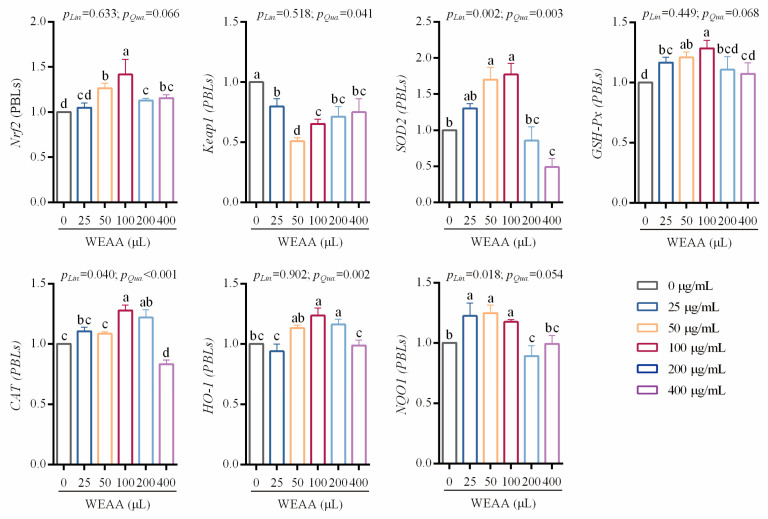
Effects of WEAA levels on the gene expression related to antioxidation in PBLs. Note: WEAA: Water extracts of *Artemisia annua* L. Different concentrations of WEAA (0, 25, 50, 100, 200, and 400 μg/mL); values are expressed as the means of 6 replicates in each group. Dose-dependent effects of WEAA (Lin: Linear; Qua: Quadratic); ^a–d^ Different letters indicate significant differences between mean values for a given indicator (*p* < 0.05), whereas the probability value of 0.05 ≤ *p* < 0.10 was considered as a tendency (±standard error).

In Figure 5, with the increase in the addition level of WEAA, the expression of *Nrf2* and *GSH-Px* in the PBLs exhibited a quadratic increasing trend (*p* = 0.066; *p* = 0.068) in the PBLs; the expression of *SOD2* and *CAT* in the PBLs showed a significant linear (*p* < 0.01; *p* < 0.01) or quadratic rise (*p* < 0.01; *p* < 0.01; *p* < 0.05; *p* < 0.01) in the PBLs; the expression of *HO-1* in the PBLs exhibited a significant quadratic rise (*p* < 0.01) and the *Keap1* expression level exhibited a quadratic decrease (*p* < 0.05) in the PBLs; the expression of *NQO1* in the PBLs showed a significant linear increase (*p* < 0.05) and/or a quadratic increasing trend (*p* = 0.054) in the PBLs.

Compared with the control group, the groups supplemented with 50–400 µg/mL of WEAA showed an increase in the *Nrf2* expression level (*p* < 0.05) in PBLs; supplementation with 25–400 µg/mL of WEAA decreased the expression of *Keap1* (*p* < 0.05) in PBLs; supplementation with 50 and 100 µg/mL of WEAA increased the expression of *SOD2* in PBLs (*p* < 0.05); supplementation with 25–100 µg/mL of WEAA increased the *GSH-Px* and *NQO1* gene expression level (*p* < 0.05) in PBLs; supplementation with 100 and 200 µg/mL of WEAA increased the expression of *CAT* in PBLs, (*p* < 0.05) and supplementation with 50 and 200 µg/mL of WEAA increased the expression of *HO-1* in PBLs (*p* < 0.05).

### 3.6. Effects of WEAA on Antioxidative Indexes in the Culture Medium of ML385-Blocked PBLs

Table 6 illustrates the effects of WEAA on the antioxidant indices in the culture medium of ML385-blocked PBLs. The results of the main effect analysis indicate that ML385 significantly reduced the T-SOD, GSH-Px, and CAT activity in the PBL culture medium (*p* < 0.05); however, WEAA significantly enhanced the levels of T-AOC, GSH-Px, CAT (*p* < 0.05), and T-SOD (*p* < 0.01) in the PBL culture medium and tended to reduce the content of MDA (*p* = 0.064). The results of the interaction effect analysis revealed that the interaction effect of WEAA and ML385 on the GSH-Px and CAT activity in the PBL culture medium was significant (*p* < 0.05).

Compared with the control group, the ML385(−)/WEAA(+) group demonstrated an increase in the activity of T-SOD, GSH-Px, and CAT (*p* < 0.05) in PBLs; the ML385(+)/WEAA(−) group decreased the T-SOD and CAT activity (*p* < 0.05), and the ML385(+)/WEAA(+) group decreased CAT activity (*p* < 0.05) in PBLs.

### 3.7. Effects of WEAA on Gene Expression of Keap1/Nrf2 Signaling Pathway in ML385-Blocked PBLs

**Figure 6:**
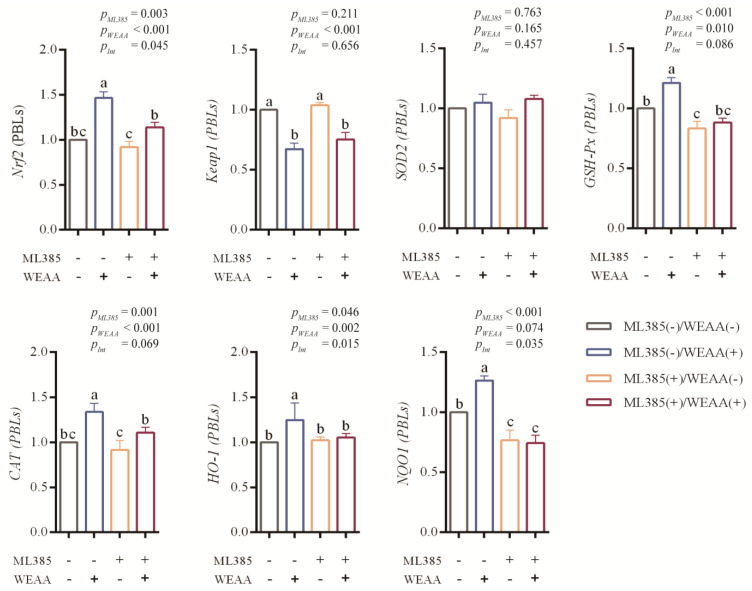
Effects of WEAA on the gene expression of Keap1/Nrf2 signaling pathway in ML385-blocked PBLs. Int: Interaction effect. Note: WEAA: Water extracts of *Artemisia annua* L. ML385: Nrf2 inhibitor. The PBLs were divided into four treatment groups: ML385(−)/WEAA(−) group, ML385(−)/WEAA(+) group, ML385(+)/WEAA(−) group and ML385(+)/WEAA(+) group; values are expressed as the means of 6 replicates in each group. ^a–c^ Different letters indicate significant differences between mean values for a given indicator (*p* < 0.05), whereas the probability value of 0.05 ≤ *p* < 0.10 was considered as a tendency (±standard error).

In Figure 6, the results of the main effect analysis demonstrate that ML385 significantly reduced the *Nrf2*, *GSH-Px*, *CAT* and *NQO1* gene expression levels (*p* < 0.01) and the expression of *HO-1* (*p* < 0.05) in the PBLs; however, WEAA significantly enhanced the expression of *Nrf2*, *CAT* and *HO-1* (*p* < 0.01), and the *GSH-Px* gene expression levels (*p* < 0.05) in the PBLs and tended to increase the gene expression of *NQO1* (*p* = 0.074). WEAA significantly decreased the expression of *Keap1* (*p* < 0.01) in the PBLs. The results of the interaction effect analysis revealed that the interaction effect of WEAA and ML385 on the gene expression of *Nrf2*, *HO-1,* and *NQO1* was significant (*p* < 0.05) and that on the expression of *GSH-Px* and *CAT* showed a significant tendency (*p* = 0.069; *p* = 0.086) in the PBLs.

Compared with the control group, the ML385(−)/WEAA(+) group showed an increase in the *Nrf2*, *GSH-Px*, *CAT*, *HO-1* and *NQO1* gene expression levels in PBLs (*p* < 0.05); the ML385(+)/WEAA(−) group decreased the *GSH-Px* and *NQO1* gene expression levels (*p* < 0.05), and the ML385(+)/WEAA(+) group decreased the expression of *NQO1* (*p* < 0.05) in PBLs.

## 4. Discussion

*Artemisia* plants, owing to their abundant bioactive components, including polysaccharides, flavonoid compounds, phenolic substances, and coumarins [22,23,24], have been extensively investigated for their nutritional and medicinal values in relevant domains. Among the numerous *Artemisia* species, *A. annua* and its extracts are rich in terpenoid compounds, and their antimalarial activity is globally renowned. They demonstrate remarkable traits in the medical field, encompassing antimalarial, antioxidant, antiviral, and immunomodulatory activity [15,25,26]. Upon an in-depth analysis at the molecular mechanism level, a variety of bioactive components in *A. annua* were found to exert antioxidant effects through intricate signal transduction pathways. For instance, polysaccharides, flavonoids, and terpenoids may directly or indirectly activate signaling pathways such as Nrf2, PI3K-Akt, MAPK, and NF-κB; upregulate the expression of downstream antioxidant enzyme genes; and promote the synthesis of antioxidant enzymes like SOD, GSH-Px, and CAT, thereby enhancing the organism’s antioxidant defense capacity. However, there has been limited systematic research conducted on the effects of WEAA on antioxidant function in ruminants, especially sheep. To gain a preliminary understanding of the antioxidant mechanism of WEAA, experiments were carried out in sheep. The current study found that different doses of WEAA in the diet significantly enhanced the activity of antioxidant enzymes in sheep serum liver and spleen and significantly decreased the content of MDA. Previous studies demonstrated that the addition of *A. annua* leaves to the diet of laying hens significantly enhanced GSH-Px activity in plasma and reduced the generation of MDA [27]. Recently, Cui et al. (2024) [28] documented that dietary supplementation with *A. annua* in geese could enhance antioxidant indices (T-AOC, SOD, GSH-Px, and CAT) in serum and jejunal tissues. Notably, the study also observed a significant decrease in MDA levels, indicating improved redox homeostasis in the experimental subjects. Additionally, there were also studies indicating that *A. ordosica* polysaccharides (AOP) significantly increased the activity of T-SOD, GSH-Px, and CAT in goat serum [29]. Wan et al. (2016) [30] reported that *A. annua* leaves with high total phenolic (44.24 ± 2.12 mg GAE/g) and flavonoid (27.80 ± 2.25 mg RE/g) contents significantly enhanced liver antioxidant enzyme activity (GSH-Px, CAT) and reduced MDA levels in broilers, highlighting the positive role of phenolic and flavonoid compounds in improving liver antioxidant capacity. Previously, our study found that WEAA was rich in organic acids, polysaccharides, flavonoids, and terpenoid compounds. Given the antioxidant potential of these components, we speculate that WEAA may exert similar antioxidant effects by activating the Keap1/Nrf2 signaling pathway or modulating redox-related enzymes. However, this hypothesis faces challenges and limitations: currently, WEAA standards lack uniformity; its active components remain incompletely elucidated, and systematic discussions on the bioavailability of active ingredients and toxicological evaluations are absent. Subsequent research will focus on the characterization and safety assessment of WEAA, thereby fortifying its application foundation in sheep breeding.

Extensive research has demonstrated that plant active substances can improve the activity of antioxidant enzymes through the Nrf2 signaling pathway and enhance the antioxidant capacity of animals. As a central hub governing cellular redox homeostasis, the activation of Nrf2 hinges on oxidation-sensitive conformational alterations in the Keap1 protein processes, whereby its Kelch domain mediates ubiquitination-dependent degradation of Nrf2 through recognition of ETGE/DLG motifs within the transcription factor. This repressive interaction is abrogated by covalent modification of critical cysteine residues (e.g., Cys151) in Keap1, a regulatory event often induced by bioactive phytoconstituents. For example, when weaning piglets were fed a diet containing *A. annua* treated with enzymes at different doses, the activity of GSH-Px and T-SOD in the serum and small intestine was increased, the concentration of MDA and 8-OHdG was reduced, and the gene and protein expression levels of *Nrf2* and *HO-1* were upregulated [31]. Studies also found that, in the water-immersion restraint stress model, pretreatment with *Artemisia* isopropanol extract (mainly flavonoids) promoted Nrf2 nuclear translocation in rat gastric mucosa, upregulated nuclear *Nrf2* expression, activated the Nrf2/HO-1 pathway, and thereby enhanced antioxidant enzyme activity, reduced MDA production, and alleviated oxidative damage [32]. Uy et al. (2025) [33] showed that extracts from the *Artemisia* genus significantly upregulated the expression and nuclear translocation of *Nrf2* protein in HT22 cells, enabling it to bind to ARE, thereby promoting the transcription and synthesis of HO-1. HO-1 catalyzes the decomposition of heme to produce bilirubin, which has a direct free radical scavenging effect [34] and can reduce MDA levels. Shi et al. (2022) [35] also found that the flavonoids of *A. ordosica* could significantly alleviate the decreased activity of antioxidant enzymes (SOD, CAT, GSH-Px) and the excessive generation of MDA in the LPS-induced model and that the underlying mechanism involves the compensatory upregulation of Keap1 mRNA and the transcriptional activation of Nrf2-downstream antioxidant enzyme genes—phenomena collectively implying a negative feedback regulatory feature within the Nrf2 signaling axis. Nrf2 is a key regulator of intracellular antioxidative stress response and plays a key role in maintaining intracellular REDOX homeostasis. After dissociation from Keap1, Nrf2 enters the nucleus and binds to ARE, promoting the transcription of cellular protective factors and thereby upregulating the expression of related antioxidant genes. This process leads to a significant increase in the intracellular activity of antioxidant enzymes, thereby eliminating free radicals and reactive oxygen species and resisting oxidative damage [18]. Moreover, the activation of the Nrf2-ARE signaling pathway not only has the role of antioxidative stress but also can enhance the immune response of the organism [19]. Therefore, the Keap1/Nrf2 signaling pathway, as a key protective pathway, has significant biological importance for the immune and antioxidant functions of the organism. In our experiment, feeding different doses of WEAA to sheep significantly enhanced the *Nrf2*, *SOD2*, *GSH-Px*, *CAT,* and *NQO1* gene expression levels in the liver and spleen tissue of sheep. In the relevant literature, substantial evidence indicates that various *Artemisia* plants such as *A. ordosica*, *A. annua,* and *A. argyi* have positive bidirectional regulatory effects on animals in challenge models and in regular feeding experiments, providing an important basis for an in-depth understanding of their functional characteristics under different body conditions [35,36,37]. These studies indicate that most plant species of this genus have positive effects on animal organisms or immune cells and may exert antioxidant regulatory effects through their rich bioactive components and related mechanisms.

However, further exploration is necessary to understand whether WEAA effects are mediated through Nrf2 signaling. After confirming the antioxidant effect of WEAA in sheep, we further evaluated the effect of WEAA by using sheep peripheral lymphocyte culture in combination with ML385 blockade, a highly specific Nrf2 inhibitor. Although lymphocytes may not perfectly replicate the state of the animal body, as one of the main immune cells in the body, lymphocytes have a significant core position in immune regulation and are often used to study many aspects such as immune response in cell culture system [17]. Firstly, the in vitro effects of different gradients of WEAA on the measured parameters in the culture system of PBLs were performed. The results showed that 50–200 μg/mL WEAA could effectively enhance the levels of T-AOC, T-SOD, CAT and GSH-Px. At the same time, the expression levels of *Nrf2* and a series of downstream target genes (*SOD2*, *CAT*, *GSH-Px*, *HO-1* and *NQO1*) were significantly up-regulated. This observation is consistent with the previous assessment of antioxidant findings in sheep serum, liver, and spleen, where a better positive effect manifested at the dose of 100 μg/mL; therefore, this dose group was selected for the inhibition test.

In a study on the construction of HepG2 cell experimental model to explore the protective mechanism of WEAA on the liver, it was observed that WEAA could significantly increase the activity levels of SOD and GSH-Px and effectively reduce the level of ROS [28]. Reddy et al. (2014) [38] studied the protective effect of *A. annua* extract in a D-galactose-induced oxidative stress model in mice; their findings indicated that *A. annua* extract effectively lowered MDA and 8-OHdG concentrations in both blood serum and liver tissue while also upregulating *NQO1* expression in organs, including the kidneys, stomach, and intestine tissues. It was confirmed that the protective effects of *A. annua* extract against lipid peroxidation and DNA damage may be due to its role in modulating antioxidant-related enzymes like NQO1, which is commonly involved in cellular antioxidant defenses and is regulated via the Nrf2 signaling cascade. These findings demonstrated the potential of natural active components of *A. annua*, such as polysaccharides, flavonoids, terpenoids, and coumarin, in the antioxidant function through the Nrf2 pathway. To better understand the mechanism of this process, Nrf2-specific inhibitors were used in the study. ML385 is a novel and specific inhibitor of Nrf2. Studies have shown that ML385 inhibits the binding of Nrf2 to ARE mainly by directly binding to the Neh1 DNA-binding region [39]. As a specific sequence in the promoter region of downstream genes regulated by Nrf2 in ARE, the binding effect of ML385 disrupts the transcriptional process of Nrf2 to activate a series of antioxidant and detoxification genes, thereby downregulating the expression of *Nrf2* target genes. Therefore, ML385 has potential application prospects in the treatment of various diseases. For example, researchers explored the mechanism by which AOP regulated the antioxidant capacity of PBLs in broiler chickens and found that inhibiting the expression of *Nrf2* with ML385 could significantly weaken the protective effect of AOP against oxidative stress and block the Keap1/ Nrf2 pathway [8]. Similarly, in our experiment, when ML385 was used alone, the activity of T-SOD and CAT and the gene expression levels of *Nrf2* and *NQO1* in PBLs were significantly reduced, but no notable disparities were detected in the genes associated with any other pathway-related genes. This indicated that the blocker inhibited the activation of Keap1/Nrf2 signaling pathway and its downstream gene (*NQO1*), which in turn reduced the production of downstream factors. When ML385 was used with WEAA, it inhibited the antioxidant activity of WEAA, suggesting that part of the active effect exerted by WEAA involves the activation mechanism of Nrf2. It further confirmed the crucial mediating role of the Keap1/Nrf2 pathway in the regulation of antioxidant function by WEAA, which provides strong validation for the research mechanism proposed earlier. In research related to *A. annua*, researchers have discovered compounds, mainly including polysaccharides, flavonoids, terpenoids, and coumarins [22,40,41,42], which are likely to play a key role in its activity. Overall, WEAA showed great potential in enhancing the antioxidant function of sheep under conventional feeding conditions, and WEAA manifested notable antioxidant function through the activation of the Keap1/Nrf2 signaling cascade.

## 5. Conclusions

To summarize, WEAA showed antioxidant capacity both in vivo and in vitro. Specifically, dietary supplementation of WEAA increased the activity of T-AOC, CAT, and GSH-Px, while reducing MDA levels in serum, liver, and spleen tissues and activating the TLR4/NF-κB pathway in lambs. In vitro validation further showed that WEAA promoted Nrf2 nuclear translocation, thereby driving the transcription of antioxidant genes (e.g., *SOD2*, *GSH-Px*, *CAT*, *NQO1*) and subsequent elevation of enzyme activity, thus enhancing antioxidant activity. These findings highlight WEAA as a promising natural feed additive for mutton sheep farming. However, further studies are warranted to elucidate the specific contributions of individual bioactive compounds in WEAA to these observed effects at the molecular level.

## Figures and Tables

**Table 1:** Composition and nutrient levels of the basal diet (%, air-dry basis).

Item	Content
Ingredients	
Alfalfa	16.25
Corn straw grass	14.00
Oat grass	24.75
Corn	23.25
Soybean meal	10.95
Wheat bran	4.25
Corn germ meal	1.95
Soybean oil	1.10
Limestone	1.10
Calcium phosphate dibasic	0.70
Salt	0.40
Sodium bicarbonate	0.80
Premix ^1^	0.50
Total	100.00
Nutrient levels ^2^	
Digestible energy (MJ/kg)	12.01
Dry matter	89.89
Crude protein	15.80
Neutral detergent fiber	40.80
Acid detergent fiber	26.24
Calcium	1.08
Phosphorus	0.40

^1^ The premix provided the following nutrient content for one kilogram of diet: vitamin A, 6000 IU; vitamin D_3_, 2500 IU; vitamin E, 12.5 IU; vitamin K_3_, 31.8 mg; vitamin B_1_, 0.035; vitamin B_2_, 8.5 mg; vitamin B_6_, 0.9 mg; nicotinic acid, 22 mg; D-pantothenic acid, 17 mg; vitamin B_12_, 0.03 mg; biotin, 0.14 mg; folic acid, 1.5 mg; Fe, 0.04 g; Cu, 0.008 g; Zn, 0.05 g; Mn, 0.03 g; I, 0.3 mg; Se, 0.3 mg; Co, 0.25 mg. ^2^ Digestible energy was a calculated value, while the others were measured values.

**Table 2:** Primers for target genes used in qRT-PCR.

Gene ^1^	Sequence (5′- > 3′) ^2^	GenBank No.	Length/bp
*β-actin*	F-ACAATGTGGCCGAGGACTTT	NM_001009784.3	278
	R-GCCGTGATGGCTGACCATTC		
*GAPDH*	F-TTATGACCACTGTCCACGCC	NM_001190390.1	216
	R-TCAGATCCACAACGGACACG		
*Nrf2*	F-TGTGGAGGAGTTCAACGAGC	XM_004004557.1	88
	R-CGCCGCCATCTTGTTCTTG		
*Keap1*	F-TTCAACAGCGAAAGTCAGGC	XM_027969637.2	157
	R-TGCGTAGCCTCCGATACTCT		
*SOD2*	F-AAACCGTCAGCCTTACACC	NM_001280703.1	116
	R-ACAAGCCACGCTCAGAAAC		
*GSH-Px*	F-TGGTCGTACTCGGCTTCCC	XM_004018462.1	163
	R-AGCGGATGCGCCTTCTCG		
*CAT*	F-GAGCCCACCTGCAAAGTTCT	XM_004016396	148
	R-CTCCTACTGGATTACCGGCG		
*HO-1*	F-CGATGGGTCCTCACACTCAG	XM_027967703.2	74
	R-CACACTCGCATTCACATGGC		
*NQO1*	F-CTCTGGCCAATTCAGAGTGG	XM_004015102.5	296
	R-TCCATTGGGATGGACTTGCC		

^1^ β-actin, beta-actin; GAPDH, Glyceraldehyde-3-phosphate dehydrogenase; Nrf2, Nuclear factor erythroid 2-related factor 2; Keap1, kelch like ECH associated protein 1; SOD2, Superoxide dismutase 2; CAT, Catalase; GSH-Px, Glutathione peroxidase; HO-1, Heme oxygenase 1; NQO1, NAD(P)H quinone dehydrogenase 1. ^2^ F: forward primer; R: reverse primer.

**Table 3:** Effects of dietary WEAA on antioxidant indexes in the serum of sheep.

Item ^1^	WEAA Supplemental Level, mg/kg				SEM	*p*-Value ^2^	
	0	500	1000	1500		Linear	Quadratic
Day 0							
T-AOC, mM	0.68	0.67	0.69	0.70	0.01	0.256	0.398
T-SOD, U/mL	83.76	82.48	84.75	82.71	1.57	0.952	0.991
GSH-Px, U/mL	81.31	81.48	81.22	81.57	3.18	0.936	0.995
CAT, U/mL	1.50	1.74	1.65	1.48	0.15	0.912	0.747
MDA, nmol/mL	2.13	2.13	2.16	2.06	0.10	0.838	0.951
Day 30							
T-AOC, mM	0.64	0.66	0.63	0.67	0.01	0.617	0.837
T-SOD, U/mL	81.88	82.33	82.72	82.46	1.12	0.837	0.968
GSH-Px, U/mL	89.42 ^ab^	104.93 ^a^	80.18 ^ab^	64.58 ^b^	5.92	0.060	0.068
CAT, U/mL	1.42 ^b^	3.05 ^a^	3.15 ^a^	3.03 ^a^	0.20	0.005	0.001
MDA, nmol/mL	2.12 ^a^	1.93 ^ab^	1.71 ^b^	1.85 ^ab^	0.06	0.076	0.084
Day 60							
T-AOC, mM	0.62 ^b^	0.64 ^ab^	0.68 ^a^	0.67 ^a^	0.01	0.014	0.029
T-SOD, U/mL	89.64	90.22	91.31	89.32	0.86	0.987	0.77
GSH-Px, U/mL	93.69 ^b^	117.04 ^ab^	145.98 ^a^	129.77 ^ab^	6.60	0.017	0.016
CAT, U/mL	2.35 ^c^	3.77 ^b^	6.07 ^a^	4.10 ^b^	0.26	0.001	<0.001
MDA, nmol/mL	1.82 ^a^	1.63 ^ab^	1.50 ^b^	1.82 ^a^	0.05	0.762	0.020

^1^ WEAA: Water extracts of *Artemisia annua* L. Different concentrations of WEAA (0, 500, 1000, and 1500 mg/mL); values are expressed as the means of 8 sheep in each group. ^2^ Values within a row with different superscripts (a, b, c) differ significantly at *p* < 0.05, whereas the probability value of 0.05 ≤ *p* < 0.10 was considered as a tendency.

**Table 4:** Effects of dietary WEAA on antioxidant indexes in the spleen of sheep.

Item	WEAA Supplemental Level, mg/kg ^1^				SEM	*p*-Value ^2^	
	0	500	1000	1500		Linear	Quadratic
Liver							
T-AOC, μmol/mg protein	0.0277	0.0278	0.0308	0.0230	0.00	0.259	0.106
T-SOD, U/mg protein	1066.79	1070.69	1165.97	1075.04	19.04	0.490	0.371
GSH-Px, U/mg protein	114.81	124.79	121.88	112.65	2.75	0.709	0.207
CAT, U/mg protein	68.52 ^b^	75.75 ^a^	76.52 ^a^	70.36 ^ab^	1.25	0.581	0.018
MDA, nmol/mg protein	3.55 ^a^	2.88 ^b^	2.97 ^ab^	3.27 ^ab^	0.10	0.427	0.043
Spleen							
T-AOC, μmol/mg protein	0.0185 ^c^	0.0240 ^b^	0.0295 ^a^	0.0218 ^bc^	0.00	0.101	0.001
T-SOD, U/mg protein	542.55 ^b^	667.18 ^ab^	682.75 ^a^	653.96 ^ab^	23.19	0.092	0.056
GSH-Px, U/mg protein	157.00 ^b^	157.00 ^b^	209.23 ^a^	160.34 ^b^	4.95	0.163	0.013
CAT, U/mg protein	2.08 ^c^	2.73 ^b^	3.19 ^a^	2.81 ^ab^	0.10	0.002	<0.001
MDA, nmol/mg protein	2.40 ^a^	1.45 ^c^	1.60 ^bc^	2.26 ^ab^	0.14	0.993	0.009

^1^ WEAA: Water extracts of *Artemisia annua* L. Different concentrations of WEAA (0, 500, 1000, and 1500 mg/mL); values are expressed as the means of 8 sheep in each group. ^2^ Values within a row with different superscripts (a, b, c) differ significantly at *p* < 0.05, whereas the probability value of 0.05 ≤ *p* < 0.10 was considered as a tendency.

**Table 5:** Effects of WEAA on antioxidant indexes in the culture medium of PBLs.

Item	WEAA Supplemental Level, µg/mL ^1^						SEM	*p*-Value ^2^	
	0	25	50	100	200	400		Linear	Quadratic
T-AOC, mM	0.43 ^b^	0.42 ^b^	0.48 ^ab^	0.51 ^a^	0.53 ^a^	0.48 ^ab^	0.01	0.085	0.002
T-SOD, U/mL	38.8 ^cd^	40.78 ^c^	36.68 ^d^	48.45 ^a^	44.75 ^b^	39.00 ^cd^	1.00	0.837	0.001
GSH-Px, U/mL	42.53 ^b^	44.62 ^b^	55.95 ^a^	60.13 ^a^	58.74 ^a^	34.51 ^c^	2.53	0.118	<0.001
CAT, U/mL	0.60 ^cd^	0.78 ^bc^	0.82 ^b^	1.09 ^a^	0.63 ^bcd^	0.53 ^d^	0.05	0.078	0.030
MDA, nmol/mL	2.75 ^a^	2.32 ^ab^	2.01 ^b^	2.15 ^b^	2.22 ^b^	2.25 ^b^	0.08	0.435	0.101

^1^ WEAA: Water extracts of *Artemisia annua* L. Different concentrations of WEAA (0, 25, 50, 100, 200, and 400 μg/mL); values are expressed as the means of 6 replicates in each group. ^2^ Values within a row with different superscripts (a, b, c, d) differ significantly at *p* < 0.05, whereas the probability value of 0.05 ≤ *p* < 0.10 was considered as a tendency.

**Table 6:** Effects of WEAA on antioxidative indexes in the culture medium of ML385-blocked PBLs.

Item	ML385(−) ^1^		ML385(+)		SEM	*p*-Value		
	WEAA(−)	WEAA(+)	WEAA(−)	WEAA(+)		ML385	WEAA	WEAA × ML385
T-AOC, mM	0.40 ^ab^	0.42 ^a^	0.39 ^ab^	0.41 ^b^	0.02	0.184	0.023	0.799
T-SOD, U/mL	30.56 ^b^	37.08 ^a^	25.46 ^c^	28.19 ^bc^	1.15	<0.001	0.008	0.239
GSH-Px, U/mL	74.83 ^b^	95.98 ^a^	58.70 ^c^	59.38 ^c^	3.82	<0.001	0.031	0.03
CAT, U/mL	0.72 ^b^	1.29 ^a^	0.70 ^b^	0.68 ^b^	0.08	0.03	0.058	0.04
MDA, nmol/mL	0.90 ^ab^	0.70 ^b^	1.05 ^a^	0.94 ^ab^	0.05	0.022	0.064	0.592

^1^ WEAA: Water extracts of *Artemisia annua* L. ML385: Nrf2 inhibitor. Values are expressed as the means of 6 replicates in each group. Values within a row with different superscripts (a, b, c) differ significantly at *p* < 0.05, whereas the probability value of 0.05 ≤ *p* < 0.10 was considered as a tendency.

## Data Availability

All data are available in the tables, figures, and Supplementary Materials.

## Supplementary Materials

The following supporting information can be downloaded at: https://www.mdpi.com/article/10.3390/antiox14070885/s1, Table S1: Compound contents of WEAA (DM basis, %).
