# Supplementary material for: Study on the Regulatory Effect of Water Extract of Artemisia annua L. on Antioxidant Function of Mutton Sheep via the Keap1/Nrf2 Signaling Pathway
Source: Antioxidants (Basel). 2025 Jul 18;14(7):885. doi: 10.3390/antiox14070885 (PMC12292189; doi:10.3390/antiox14070885)
Supplement: Supplementary file 1 [file antioxidants-14-00885-s001.zip › antioxidants-3733718-supplementary.pdf]

Supplementary Table S1. Compound contents of WEAA (DM basis, %)

**Compounds**	**contents**
Organic acids and derivatives	24.61
Soluble polysaccharide	18.64
Flavonoids	9.80
Prenol lipids	7.75
Organoheterocyclic compounds	7.75
Organooxygen compounds	5.01
Nucleosides, nucleotides, and analogues	5.01
Fatty acyls	4.79
Benzene and substituted derivatives	3.87
Glycerophospholipids	2.28
Coumarins and derivatives	2.05
Cinnamic acids and derivatives	1.82
Phenols	1.60
Others	5.01
